# COVID-19 Vaccination-Induced Myopericarditis: An Imager’s Perspective

**DOI:** 10.1016/j.cjco.2022.01.007

**Published:** 2022-01-29

**Authors:** Hilary Bews, Ashley Bryson, Tessa Bortoluzzi, James W. Tam, Davinder S. Jassal

**Affiliations:** a Section of Cardiology, Department of Internal Medicine, Rady Faculty of Health Sciences, University of Manitoba, Winnipeg, Manitoba, Canada; b Institute of Cardiovascular Sciences, St. Boniface Hospital, University of Manitoba, Winnipeg, Manitoba, Canada; c Department of Radiology, St. Boniface Hospital, University of Manitoba, Winnipeg, Manitoba, Canada; d Department of Physiology and Pathophysiology, Max Rady College of Medicine, Rady Faculty of Health Sciences, University of Manitoba, Winnipeg, Manitoba, Canada

## Abstract

COVID-19 vaccine-induced myocarditis is a rare adverse event in the current pandemic. The following is a case series of 10 individuals with COVID-19 vaccine-related myocarditis confirmed by cardiac magnetic resonance imaging. In this cohort of predominantly male patients, with a mean age of 23 years, chest discomfort and positive cardiac biomarkers occurred at a median of 3 days after the second COVID-19 vaccine dose. Although systolic function was relatively preserved on noninvasive cardiac imaging, evidence was seen of delayed enhancement on cardiac magnetic resonance imaging, confirming myocarditis. As COVID-19 vaccine-induced myocarditis has a relatively benign clinical course, the benefits of vaccination still, by far, outweigh this small risk.

## Résumé

La myocardite induite par le vaccin contre la COVID-19 est un événement indésirable rare de la pandémie actuelle. Voici une série de cas de 10 individus atteints d’une myocardite liée au vaccin de la COVID-19 confirmée par l’imagerie par résonance magnétique cardiaque (RMC). Dans cette cohorte principalement composée d’hommes, dont l’âge moyen était de 23 ans, qui ont manifesté un inconfort à la poitrine et montré des biomarqueurs cardiaques positifs à une médiane de trois jours après la deuxième dose du vaccin contre la COVID-19. Bien que l’imagerie cardiaque non invasive a montré une fonction systolique relativement préservée, la RMC mettait en évidence un rehaussement tardif qui confirmait la myocardite. Puisque la myocardite induite par le vaccin contre la COVID-19 a une évolution clinique relativement bénigne, les avantages de la vaccination restent de loin supérieurs à ce risque minime.

The COVID-19 pandemic has resulted in unprecedented morbidity and mortality, and it has had economic and social impacts that will continue for decades to come. In response to the pandemic, 2 mRNA vaccines were developed, by Pfizer and Moderna, respectively, and were approved by Health Canada in December 2020. Since that time, a small number of case reports have described the rare occurrence of myocarditis after COVID-19 vaccination, raising the confirmation of this rare vaccine-related adverse event. We report a case series of 10 patients diagnosed with COVID-19 vaccine-related myocarditis by cardiac magnetic resonance (CMR) imaging.

## Case Series

All CMR studies positive for myocarditis between March and September 2021 at the St. Boniface Hospital in Winnipeg, Manitoba, Canada were evaluated. Patients were identified as having COVID-19 vaccine-induced myocarditis as adapted from the case definition and classification of the Brighton Collaboration (Pandemic Emergency Process).^1^ Additionally, all patients met the following criteria: (i) clinical syndrome consistent with myocarditis; (ii) COVID-19 vaccination within 21 days after the first dose of the vaccine or 30 days after the second dose^2^; and (iii) exclusion of other etiologies (including active severe acute respiratory syndrome coronavirus 2 [SARS-CoV-2] infection).

Between March 16, 2021 and September 3, 2021, out of 42 cases of CMR-confirmed myocarditis, a total of 10 individuals presented with COVID-19 vaccine-related myocarditis (Supplemental Table S1). The mean age of affected individuals was 23 years (range: 18-45 years). A total of 9 cases occurred in men, 2 of whom were female-to-male transgender individuals currently on testosterone therapy. The median time from vaccination to hospital presentation was 3 days (range: 2-20 days), and 90% presented after the second vaccine dose. None of the 10 individuals had a previous history of either pericarditis or myocarditis. The most common presenting symptom was pleuritic chest pain with no evidence of a pericardial rub. The mean high-sensitivity troponin T level was 846 ng/L (normal: 0-14 ng/L), and the mean C-reactive protein level was 39 mg/L (range: 3-92 mg/L; normal: < 5 mg/L). The most common finding on electrocardiography included subtle diffuse ST abnormalities. All 10 individuals had negative COVID-19 polymerase chain reaction testing on admission.

**Figure 1:**
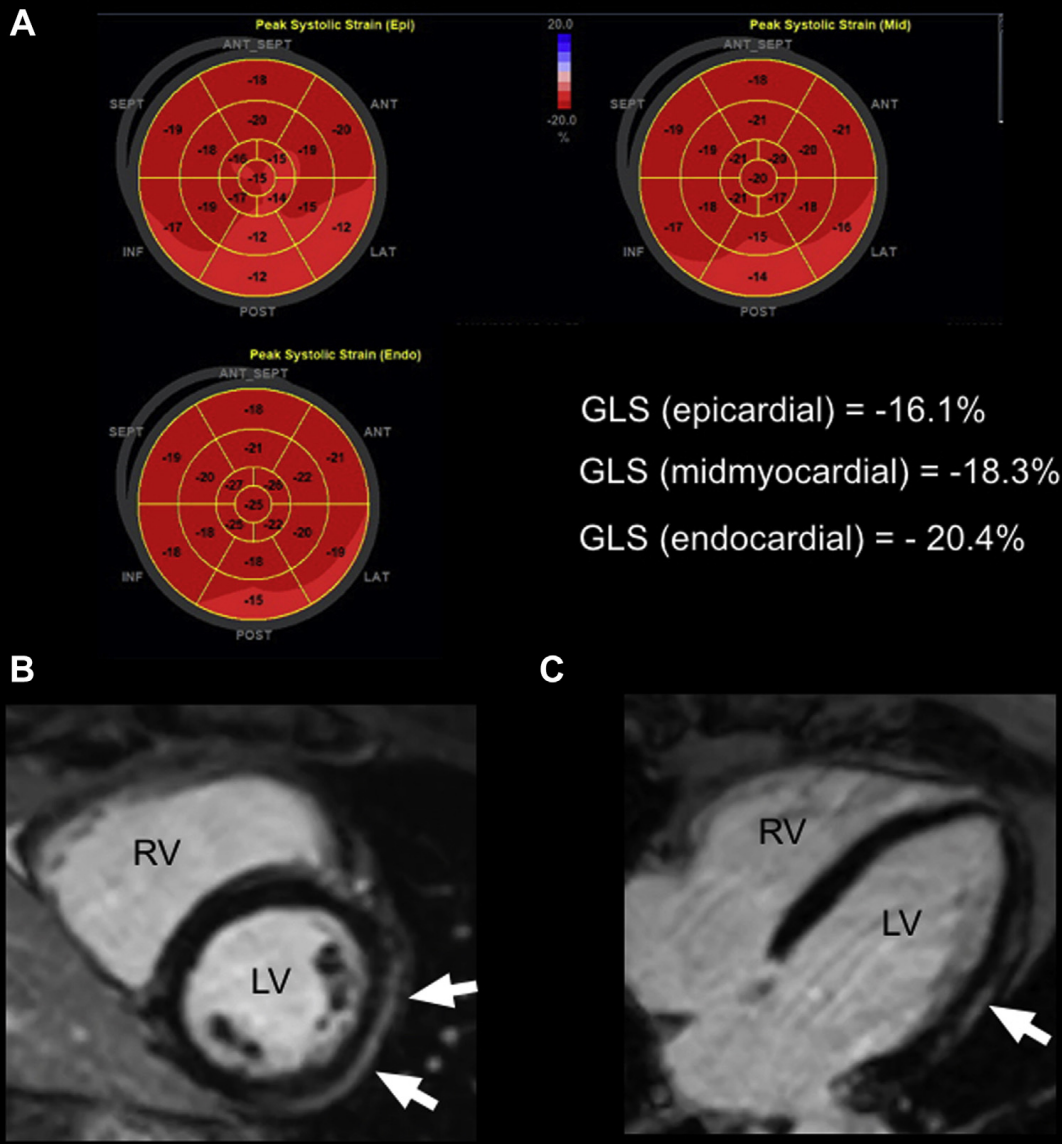
(**A**) Representative 2-dimensional strain image bullseye plots for epicardial, midmyocardial, and endocardial segments of the left ventricle. (**B**) Short-axis phase-sensitive reconstructed inversion recovery image through the mid-ventricle at the level of the papillary muscles, demonstrating subepicardial delayed enhancement (**arrows**) of the lateral (LAT) wall. (**C**) Long-axis phase-sensitive reconstructed inversion recovery image demonstrating subepicardial delayed enhancement (**arrow**) of the LAT wall. ANT, anterior; GLS, global longitudinal strain; INF, inferior; LV, left ventricle; POST, posterior; RV, right ventricle; SEPT, septal.

On transthoracic echocardiography (TTE), the mean left ventricular ejection fraction (LVEF) was 56%, with mild regional wall motion abnormalities (LVEF range: 45%-60%; Supplemental Table S2). No evidence of a pericardial effusion appeared on TTE imaging. Although the location of wall motion abnormalities did vary among cases, the basal to mid inferior and inferolateral walls were overwhelmingly affected. Diastolic function, left ventricular filling pressures, and right ventricular structure and function were normal. In a subset of 4 patients, global longitudinal strain (GLS) was performed (Fig. 1A) and was found to be reduced in a primarily epicardial distribution, with an average value of -13.7 (Supplemental Table S2).

The CMR studies were completed, on average, 10.3 days after TTE (range: 0-52 days). All patients demonstrated a preserved or borderline reduced LVEF on CMR imaging (Supplemental Table S3). On CMR imaging, regional wall motion abnormalities were observed in only 1 patient, with no evidence of a pericardial effusion. All 10 patients demonstrated late gadolinium enhancement (LGE) in a subepicardial or midmyocardial distribution, most commonly affecting the inferior, inferolateral, and lateral walls, as detailed in Supplemental Table S3 (Fig. 1, B and C). LGE of the pericardium was not observed.

All patients were admitted to the hospital for work-up of myocarditis and monitoring, with a median stay of 4.5 days (range: 3-6 days). The majority of cases were treated with ibuprofen and colchicine for acute pericarditis, and although 2 patients required additional therapy for recurrent chest pain, no adverse events, including arrhythmias, heart failure, cardiogenic shock, and death, were noted in any of the 10 cases.

## Discussion

COVID-19 vaccine-induced myocarditis is a rare adverse event in the current pandemic.^3^ Approximately 1000 cases of COVID-19 vaccine-induced myocarditis/pericarditis have been reported to the Centres for Disease Control and Prevention (CDC) Vaccine Adverse Event Reporting System (VAERS) database.^4^ This number corresponds to a rate of 12.6 cases per million doses in individuals aged 12 to 39 years after the second vaccine dose.^4^ Barda et al. compared vaccinated individuals to matched controls using data from the largest healthcare organization in Israel. They reported an increased risk of myocarditis in the vaccinated group, with a risk ratio of 3.24 (95% confidence interval 1.55 to 12.44).^5^

A number of mechanisms have been proposed to explain the finding of myocardial inflammation following COVID-19 vaccination. First, molecular mimicry between the SARS-CoV-2 spike protein, encoded for by the mRNA vaccines, and cardiac molecules may elicit an immune response in predisposed individuals.^4^ A second possible mechanism involves a generalized systemic inflammatory response triggered by antigenic mRNA, with subsequent injury to cardiomyocytes.^4^ Finally, a delayed hypersensitivity reaction has been proposed, with sensitization occurring after the first dose of COVID-19 vaccination.^6^ In one case series, the 3 patients diagnosed with myocarditis following the first COVID-19 vaccine dose had all recovered from prior natural SARS-CoV-2 infection, suggesting an alternate sensitization event.^6^ Of note, our patient who presented after the first vaccine dose did not have a previously documented SARS-CoV-2 infection.

Similar to previous case series, our findings confirm that COVID-19 vaccine-related myocarditis disproportionately affects young men, primarily after the second vaccine dose.^4^^,^^6^ In phase I/II studies, a stronger immunologic response was observed after the second vaccine dose, which may explain this finding.^7^ The overrepresentation of male cases likely relates to a complex interplay between sex differences in hormonal factors, including a role of testosterone in the inhibition of anti-inflammatory cells and stimulation of a Th1 immune response.^4^ The fact that 2 of the patients in our case series are transgender female-to-male patients who were on testosterone therapy supports this theory.

The echocardiographic and CMR findings of a normal or borderline reduced LVEF in our case series were consistent with previous reports.^8^ Of interest, although the majority of our patients were treated for acute pericarditis with anti-inflammatory agents and colchicine, no evidence of a pericardial effusion nor delayed enhancement of the pericardium appeared on TTE or CMR imaging. Despite the observation of mild regional wall motion abnormalities affecting the mid to basal inferior and inferolateral walls on TTE in the acute setting, most patients did not demonstrate wall motion abnormalities on CMR imaging. This may be, in part, due to the delay in CMR imaging, to approximately 10 days after the acute presentation. All of our patients demonstrated LGE, primarily affecting the inferior, inferolateral, and lateral walls. This distribution coincides with findings reported by Shaw et al.^8^ In addition to CMR, we report novel abnormal epicardial GLS values in all 3 patients who had this parameter measured, suggesting that GLS may be an additional noninvasive tool for the identification of COVID-19 vaccine-related myocarditis.

## Conclusion

Our study describes 10 cases of myocarditis identified using CMR imaging following COVID-19 vaccination. In this case series, young men were more commonly affected, particularly after the second mRNA vaccine dose. As COVID-19 vaccine-induced myocarditis has a relatively benign clinical course, the benefits of vaccination still, by far, outweigh this small risk.

**Novel Teaching Points**

• Myocarditis is a rare side effect following COVID-19 vaccination, overwhelmingly affecting young men after the second mRNA vaccine dose.
• The clinical courses of our 10 patients were benign.
• All patients demonstrated LGE on CMR imaging, which most commonly affected the inferior, inferolateral, and lateral walls in a subepicardial or mid-myocardial distribution.
• Abnormal epicardial global longitudinal strain by echocardiography may be an additional noninvasive tool to identify COVID-19 vaccine-related myocarditis.
