# Supplementary material for: COVID-19 Vaccination-Induced Myopericarditis: An Imager’s Perspective
Source: CJC Open. 2022 Jan 29;4(5):497–500. doi: 10.1016/j.cjco.2022.01.007 (PMC8800170; doi:10.1016/j.cjco.2022.01.007)
Supplement: Supplementary Tables S1-S3 [file mmc1.pdf]

Supplemental Table S1: Clinical features, presenting symptoms, investigations and outcomes of the 10 patients with COVID-19 vaccine related myocarditis.

	Age, Sex	Past Medical History	COVID Vaccinations	Time from Vaccination to Hospital Presentation (days)	Presenting Symptoms	ECG Changes	Peak hsTnT (ng/L)	CRP (mg/L)	Treatment	Hospital Stay (days)	Adverse Events
1	21F	Irritable bowel syndrome	Pfizer<br>Pfizer	3	Sharp central chest pain, radiation to back	Sinus tachycardia	408	3	Ibuprofen	6	Restarted on ibuprofen for recurrent chest pain
2	45M	ADHD, depression, anxiety, irritable bowel syndrome	AstraZeneca<br>Pfizer	4	Shortness of breath with intermittent burning central chest pain	Sinus rhythm with minimal diffuse ST elevation and PR depression	207	8	Colchicine, metoprolol, aspirin	5	None
3	31M	Depression	Pfizer<br>Moderna	20	Central chest heaviness, radiation to bilateral shoulders	Sinus rhythm with inferior T wave inversion, mild diffuse PR depression	367	7	Ibuprofen and colchicine	4	Ibuprofen dose increased for recurrent chest pain
4	21M	None	Pfizer<br>Moderna	3	Central chest pressure, worse with activity	Sinus rhythm with diffuse ST elevation in anterolateral and inferior leads, ST depression in aVR	1760	25	Ibuprofen and colchicine	3	None

5	31M	None	Pfizer Moderna	4	Central chest heaviness, radiation to bilateral arms. Associated palpitations, emesis and mild shortness of breath.	Sinus rhythm with diffuse ST elevation	1916	N/A	Ibuprofen and colchicine	3	None
6	30M	Female to male transition on testosterone	Pfizer Moderna	3	Aching chest pain, radiation to back and shoulders. Associated diaphoresis, weakness, nausea and diarrhea	Sinus rhythm with early repolarization	404	13	Colchicine	5	None
7	22M	None	Moderna Moderna	3	Fever, chills and diffuse myalgias since vaccination. Burning, central chest pain peaking on day of presentation	Sinus rhythm with mild diffuse ST elevation and PR depression, ST depression in aVR	232	91	None	3	None
8	23M	Female to male transition on testosterone	Pfizer Moderna	2	Sharp, pleuritic chest pain. Associated rhinitis, pharyngitis, headache, nausea and body aches	Sinus rhythm with diffuse ST elevation and mild PR depression, ST depression in aVR	2007	92	Ibuprofen and colchicine	5	None
9	18M	None	Pfizer Moderna	2	Central chest tightness, radiation to	Sinus rhythm with early repolarization	547	35	Ibuprofen and colchicine	3	None

					back, worse with inspiration						
10	18M	None	Pfizer Pfizer	3	Chest pain, worse when supine	Sinus tachycardia with diffuse ST elevation and PR depression	612	78	Ibuprofen	6	None

ADHD, Attention deficit hyperactivity disorder; CRP, C-reactive protein; ECG, Electrocardiogram; hsTnT, High sensitivity troponin T.

Supplemental Table S2: Echocardiographic findings for 10 patients with COVID-19 vaccine induced myocarditis.

	LVEDD (cm)	LVEF (%)	Diastolic Function	Left-sided Filling pressures	RV size	RV function	RWMA	Endocardial GLS	Mid-myocardial GLS	Epicardial GLS
1	4.3	60	Normal	Normal	Normal	Normal	Basal anterior and basal lateral walls hypokinetic			
2	4.4	55	Mild dysfunction	Normal	Normal	Normal	Basal to mid anterior and basal lateral walls hypokinetic	-17.8	-15.7	-14
3	4.7	56	Normal	Normal	Normal	Normal	Basal inferolateral and basal inferior walls hypokinetic	-17.4		
4	4.7	45	Moderate dysfunction	High	Normal	Normal	Global hypokinesis, worst contraction of the basal to mid inferior and mid inferolateral walls			
5	5.1	58	Normal	Normal	Normal	Normal	Basal inferior and mid inferolateral walls			
6	4.6	60	Normal	Normal	Normal	Normal	None	-20.4	-18.3	-16.1
7	4.6	57	Normal	Normal	Normal	Normal	Basal to mid inferior wall hypokinetic	-15.5	-13.5	-11
8	4.4	60	Normal	Normal	Normal	Normal	None			

9	4.7	58	Normal	Normal	Normal	Normal	Basal inferior and inferoseptal walls hypokinetic			
10	No TTE performed									

GLS, Global longitudinal strain; LVEDD, Left ventricular end diastolic dimension; LVEF; Left ventricular ejection fraction; RV, Right ventricle; RWMA, Regional wall motion abnormalities; TTE, transthoracic echocardiography.

Supplemental Table S3: Cardiac magnetic resonance imaging (CMR) findings in 10 patients with COVID-19 vaccine related myocarditis.

	LV End Diastolic Volume (ml)	LV End Systolic Volume (ml)	LV Stroke Volume (ml)	LV Ejection Fraction (%)	Wall Motion Abnormality	Pericardial Effusion	Delayed Late Gadolinium Enhancement Location
1	107	41	66	62	No	No	Sub-epicardial to mid-myocardial, involving the basal inferolateral wall
2	138	67	71	51	No	No	Sub-epicardial, involving lateral wall of the mid chamber
3	146	63	83	57	No	No	Sub-epicardial, involving the basal to mid lateral wall and basal to mid inferior wall
4	149	71	77	52	No	No	Mid-myocardial and epicardial, involving the basal lateral and inferior wall. Sub-epicardial, involving the inferolateral wall at the mid cavitary level
5	176	89	88	50	Moderate hypokinesis of the mid to distal lateral wall	No	Mid-myocardial, involving the mid to distal lateral wall

6	140	51	89	64	No	No	Mid-myocardial, involving the mid inferior septal and mid inferior regions. Sub-epicardial, involving the mid lateral wall
7	132	48	84	64	No	No	Sub-epicardial, involving the mid lateral wall
8	127	50	77	61	No	No	Sub-epicardial, involving the mid to distal lateral wall
9	155	63	93	60	No	No	Mid-myocardial, involving the mid lateral wall
10	149	53	96	64	No	No	Sub-epicardial, involving the mid to distal lateral wall

LV, left ventricle.
